# Simultaneous influence of sympathetic autonomic stress on Schlemm’s canal, intraocular pressure and ocular circulation

**DOI:** 10.1038/s41598-019-56562-0

**Published:** 2019-12-27

**Authors:** Wei Chen, Zhiqi Chen, Yan Xiang, Chaohua Deng, Hong Zhang, Junming Wang

**Affiliations:** Department of Ophthalmology, Tongji Hospital, Tongji Medical College, Huazhong University of Science and Technology, Wuhan, China

**Keywords:** Optic nerve diseases, Tomography

## Abstract

This study aimed to investigate changes in Schlemm’s canal, intraocular pressure and ocular blood circulation following the activation of the sympathetic nervous system. Twenty healthy volunteers were enrolled in this study. The cold pressor test (CPT) was adopted. Cross-sectional area of Schlemm’s canal (SCAR), superficial and deep retinal vessel densities (s-RVD;d-RVD), pupil diameter (PD), intraocular pressure (IOP), mean ocular perfusion pressure (MOPP) and heart rate variability (HRV) were measured at three time-points: baseline (T0) and 5 min (T1) and 10 min (T2) after the CPT. After cold stimulation, LF/HF index (the ratio of low frenquency and high frenquency) increased significantly. IOP decreased from 16.9 ± 1.9 mmHg at baseline to 16.4 ± 2.7 mmHg at T1 and to 15.2 ± 2.7 mmHg at T2. The nasal cross-sectional area of SCAR (SCAR-n) increased from 6283.9 ± 2696.2 µm^2^ at baseline to 8392.9 ± 3258.7 µm^2^ at T1 and to 10422.0 ± 3643.8 µm^2^ at T2. The temporal cross-sectional area of SCAR (SCAR-t) increased from 6414.5 ± 2218.7 µm^2^ at baseline to 8610.8 ± 2317.1 µm^2^ at T1 and to 11544.0 ± 4129.2 µm^2^ at T2. The expansion of Schlemm’s canal was observed after the CPT might be caused by sympathetic nerve stimulation, subsequently leading to decreased IOP.

## Introduction

The sympathetic nervous system (SNS) is a component of the peripheral nervous system that regulates many human physiological variables including heart rate, blood pressure, respiration, digestion, and sexual arousal^1^. In the eye, the intraocular pressure and ocular blood circulation were also mainly controlled by the sympathetic nervous system^2,3^. The influence of circadian rhythm in the SNS upon intraocular pressure has been a subject of interest. It was found that excision of the superior cervical sympathetic ganglion lowered, and electrical stimulation of the sympathetic nerve trunk elevated IOP^4^. In 1898, cervical sympathectomy became a popular operation, with most glaucoma surgeons fascinated about its results^5^. However, the effect on IOP was transient after interfering with the autonomic nervous system (ANS) and the mechanism by which the SNS influences IOP remains controversial.

In general, the branches of the central retinal artery are not innervated by adrenergic fibres and the systemic control of the autonomic nervous system has a minor influence, however, there are some conflicting opinions regarding the absence of the influence of sympathetic innervation on the retina^6^. Lanigan *et al*. reported a significant association between retinal vessel calibre and sympathetic nervous stimulation^2^. After sympathetic blockage (stellate ganglion blockage, SGB), the blood flow of the optic nerve head (ONH) and the ipsilateral retina significantly increased^7^; In addition, there was a report that SGB increased the retinal circulation in patients with Bell’s palsy^8^. However, no study evaluating the effects of SNS on the ONH and the retinal vessel density has been conducted.

The increased intraocular pressure is the major risk factor of glaucoma, which is a leading cause of irreversible blindness in the world^9,10^. The insufficient retinal blood supply was also found that has a role in the development of glaucoma in recent years^11^. So, it is necessary to evaluate the effect of the autonomic nervous system upon the IOP and ocular blood supply.

Several autonomic nervous function tests are available to aid clinicians, including the hand-grip exercise, head tilt test, the cold pressor test (CPT) and the water drinking test^12,13^. The CPT remains the most well-known test for the determination of ANS integrity^14^. Numerous studies have found that the CPT induces reproducible, sympathetic nervous system (SNS) activation in normal subjects^15–17^. This study aimed to examine the variations in retinal and ONH vessel density and possible mechanisms underlying IOP changes following the activation of the SNS after the CPT.

## Results

Twenty-four participants (24 eyes) were enrolled in this study. Four participants were excluded due to unsatisfactory image quality. Finally, 20 participants (20 eyes; 11 males; 9 females) were included. Participants’ mean age was 26.75 ± 3.3 years (range 22–36, years), mean best corrected visual acuity (BCVA) was 0.98 ± 0.06 (range 0.8–1.0) and mean RE was −2.14 ± 2.01 diopter (range −5.50–0.80, diopter).

**Figure 1:**
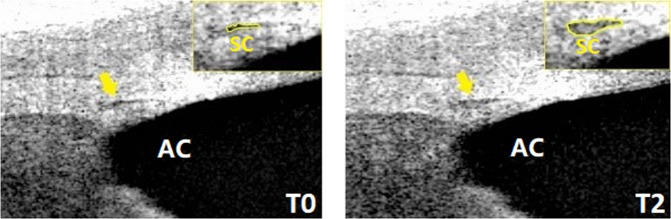
Morphology of Schlemm’s canal (yellow arrow). Before (left, T0) and after (right, T2) the cold pressor test. The SCAR increased after the CPT. SC: Schlemm’s canal; AC: anterior chamber. SCAR: Cross-sectional area of Schlemm’s canal; CPT: Cold pressor test. T0: recordings at baseline after 5 min rest; T2: recordings (6–10 min) after 1 min cold stimulation.

**Figure 2:**
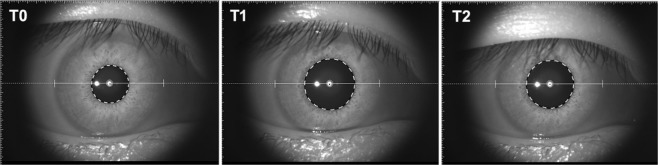
Pupil diagram during the cold pressor test. The pupil diameter increased at T1 and returned at T2.

SCAR-n, SCAR-t increased after cold stimulation (Fig. 1). SCAR-n increased from 6283.9 ± 2696.2 µm^2^ at T0 to 8392.9 ± 3258.7 µm^2^ post-CPT at T1 and increased to 10422.0 ± 3643.8 µm^2^ at 10 min post-CPT. SCAR-t increased from 6414.5 ± 2218.7 µm^2^ at T0 to 8610.8 ± 2317.1 µm^2^ post-CPT at T1 and increased to 11544.0 ± 4129.2 µm^2^ at T2. IOP changed from 16.9 ± 1.9 mmHg at T0 to 16.4 ± 2.7 mmHg at T1 and then further decreased to 15.2 ± 2.7 mmHg at T2, post-CPT. Pupil diameter increased from 5.22 ± 0.9 mm at T0 to 5.50 ± 0.9 mm at T1 and returned to 5.11 ± 0.8 mm at T2 (Fig. 2). MAP (Mean arterial pressure) increased from 91.4 ± 2.1 mmHg (T0) to 100.9 ± 2.8 mmHg (T1) and returned to 88.9 ± 2.3 mmHg (T2). MOPP (Mean ocular perfusion pressure) increased from 74.0 ± 8.4 mmHg at T0 to 84.6 ± 11.8 mmHg at T1 and then decreased to 73.3 ± 9.7 mmHg at T2, post-WDT. (Table 1). In the control group, no significant change in these parameters were found (Table 2).

**Table 1:** Parameters following cold pressor test for the experimental group.

	Pre-CPT (T0)	Post-CPT (T1)	Post-CPT (T2)	*P value*	*P’ value*
IOP (mmHg)	16.9 ± 1.9	16.4 ± 2.7	15.2 ± 2.7	0.04	<0.001
SCAR-n (μm^2^)	6283.9 ± 2696.2	8392.9 ± 3258.7	10422 ± 3643.8	0.001	<0.001
SCAR-t (μm^2^)	6414.5 ± 2218.7	8610.8 ± 2317.1	11544 ± 4129.2	0.001	<0.001
PD (mm)	5.2 ± 0.9	5.5 ± 0.9	5.1 ± 0.8	0.04	0.70
MAP (mmHg)	91.4 ± 2.1	100.0.9 ± 2.8	88.9 ± 2.3	<0.001	0.14
MOPP (mmHg)	74.0 ± 8.4	84.6 ± 11.8	73.3 ± 9.7	<0.001	0.73

P < 0.05 means the parameters changed significantly, as evaluated by the One-Way Repeated measures ANOVA (data consistence with homogeneity of variance).

*P value*: Pairwise comparison of parameters from T0 toT1; *P’ value*: Pairwise comparison of parameters from T0 to T2;

IOP: Intraocular pressure; SCAR-n: The nasal cross-sectional area of Schlemm’s canal. SCAR-t: The temporal cross-sectional area of Schlemm’s canal. PD: Pupil diameter; MAP: Mean arterial pressure; MOPP: Mean ocular perfusion pressure.

**Table 2:** Parameters following cold pressor test for the control group.

	Pre-CPT (T0)	Post-CPT (T1)	*P value*
IOP (mmHg)	16.5 ± 1.7	16.4 ± 1.8	0.640
SCAR-n (μm^2^)	5809.4 ± 2823.8	6000.1 ± 2634.7	0.392
SCAR-t (μm^2^)	6269.9 ± 2040.3	6260 ± 2052.4	0.817
PD (mm)	5.2 ± 0.9	5.5 ± 0.9	0.828
MAP (mmHg)	87.9 ± 8.3	86.8 ± 6.5	0.299
MOPP (mmHg)	47.6 ± 5.8	46.9 ± 4.7	0.348

No parameters were found changed significantly (P > 0.05), as evaluated by the Paired-Samples T test (data consistence with normally distributed). IOP: Intraocular pressure; SCAR-n: The nasal area of Schlemm’s canal. SCAR-t: The temporal area of Schlemm’s canal. PD: Pupil diameter; MAP: Mean arterial pressure; MOPP: Mean ocular perfusion pressure.

**Figure 3:**
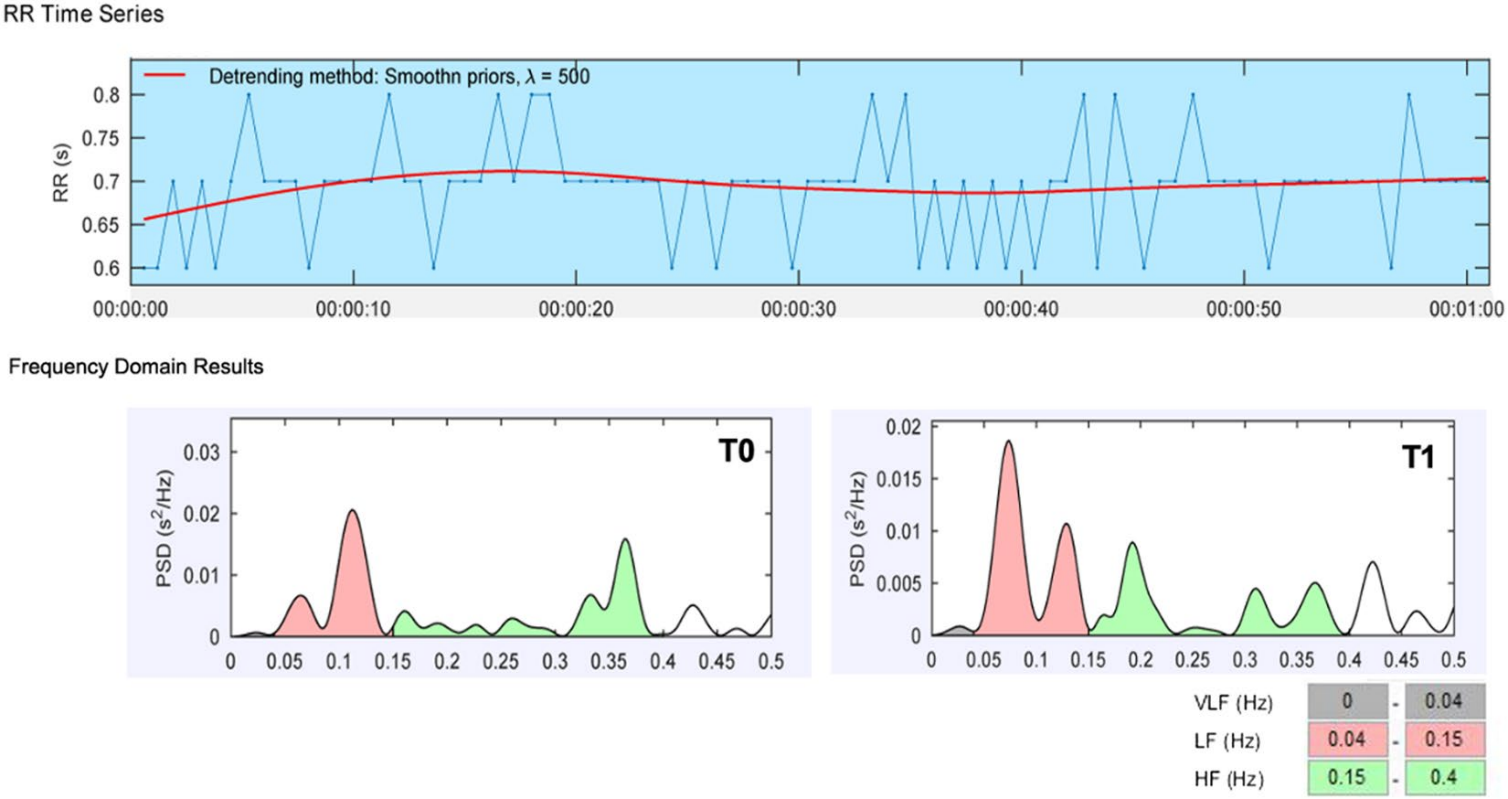
The HRV results obtained from a 3-leads ECG recording. The frequency domains are generated using Fourier transform from the recorded R–R intervals. Upper: The R–R interval curve from the 1-minute recording. Below: The frequency domain result during the CPT (T0 and T1). VLF: 0–0.04 Hz, LF: 0.04–0.15 Hz, HF:0.15–0.4 Hz. HRV: Heart rate variability; ECG: Electrocardiograph.

**Figure 4:**
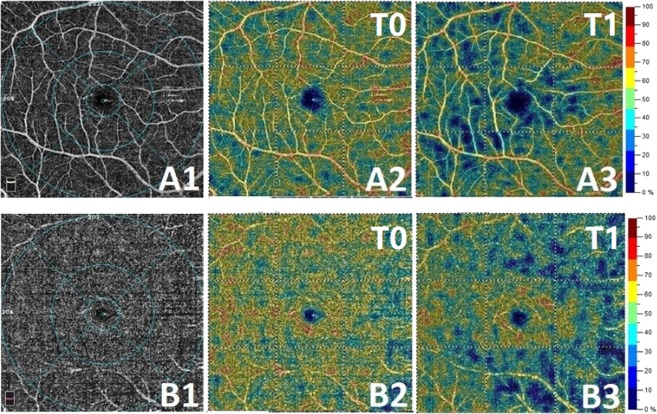
(**A**,**B**) The retinal vessel density decreased significantly at T1 after cold stimulation. A1: the diagram of OCTA in superficial retinal layer; A2: the diagram of superficial retinal vessel density at T0; A3: the diagram of superficial retinal vessel density at T1 after CPT; B1: the diagram of OCTA in deep retinal layer; B2: the diagram of deep retinal vessel density at T0; B3: the diagram of deep retinal vessel density at T1. CPT: Cold pressor test. OCTA: Optic Coherence Tomography Angiography.

After cold stimulation, LF/HF increased from 0.53 ± 0.24 at T0 to 0.82 ± 0.25 and decreased to 0.59 ± 0.24 (Fig. 3). Superficial retinal and deep vessel densities across four quadrants (inferior, superior, nasal, and temporal) decreased at T1 after CPT and then returned to baseline at T2 (Fig. 4A,B).

**Figure 5:**
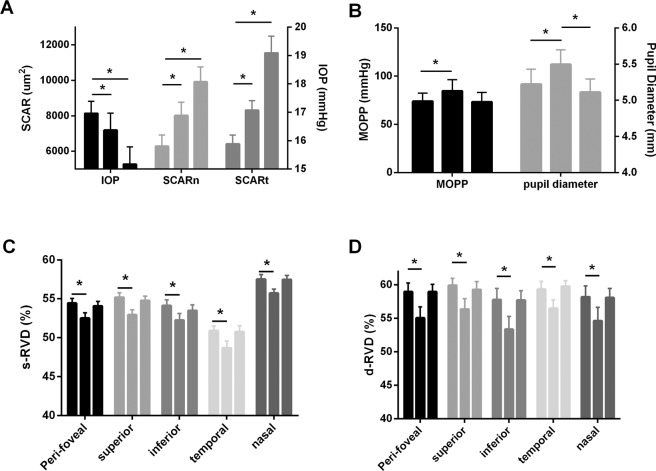
(**A**–**D**) One-way repeated measures ANOVA of SCAR-n, SCAR-t, IOP, MOPP, PD, s-RVD and d-RVD at three time-points (T0, T1, T2) during cold stimulation. The asterisk indicates statistically significant differences (P < 0.05). ANOVA: Analysis of Variance; SCAR-n: the nasal cross-sectional area of Schlemm’s canal; SCAR-t the temporal cross-sectional area of Schlemm’s canal; IOP: intraocular pressure; MOPP: mean ocular perfusion pressure; PD: pupil diameter; s-RVD: the superficial retinal network; d-RVD: the retinal deep capillary network.

The significant differences were revealed by the one-way repeated measures ANOVA test. The SCAR (nasal and temporal) significantly increased from T0 to T2; MAP and MOPP significantly increased from T0 to T1 and returned to baseline at T2. The IOP significantly decreased from T0 to T2; the LF/HF significantly increased from T0 to T1 and returned to baseline at T2. The pupil diameter significantly increased in T1 and returned to baseline in T2. Both of s-RVD and d-RVD significantly decreased at T1 and returned to baseline in T2. (Fig. 5A–D). There were no significant differences in TM-thickness and ODVD (optic disc vessel density) at any timepoints.

**Figure 6:**
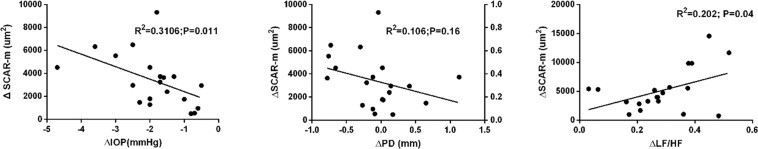
(**A–C**) The linear regression analyses of ΔSCAR-m, ΔIOP, ΔPD and ΔLF/HF. There is significant correlation of ΔSCAR-m and ΔIOP, ΔSCAR and ΔLF/HF, but not in ΔSCAR and ΔPD. ΔSCAR-m: The variation of mean value of SCAR in nasal and temporal from T0 to T2; ΔIOP: the variation of IOP from T0 to T2. ΔPD: the variation of pupil diameter from T0 to T2; ΔLF/HF: the variation of LF/HF from T0 to T2.

A significant correlation was found between ΔSCAR-m (The variation of mean value of SCAR in nasal and temporal from T0 to T2) and ΔIOP (The variation of IOP from T0 to T2; R^2^ = 0.3106, P = 0.011), and also found between ΔSCAR-m and ΔLF/HF (The variation of LF/HF from T0 to T2; R^2^ = 0.202, P = 0.04), but not in ΔSCAR-m and ΔPupil diameter (The variation of LF/HF from T0 to T2; R^2^ = 0.013, P = 0.63, Fig. 6A–C).

## Discussion

The cold pressor test was first described by Hines and Brown in 1932, which was designed to measure the reaction of the blood vessels to a standard stimulus^18^. Increased BP reaction to CPT has been associated with a higher risk of hypertension^19^. Fremann *et al*. found that the response will promote an increase of 15–20 mmHg and 10–15 mmHg in systolic and diastolic blood pressure, respectively^15^. In our study, MAP and MOPP were also found to increase obviously during the CPT. The release of norepinephrine and epinephrine, the activation of β-adrenergic receptors and α2-adrenergic activity were validated after the cold stimulation. The activation of the peripheral sympathetic nervous system during the CPT is considered as the major mechanisms mediating the vasoconstrictive response^20,21^.

Heart rate variability (HRV) is the physiological variation between the R-R beat, which could provide real-time information of the peripheral autonomic nervous system non-invasively. HRV was used in our study to evaluate the SNS activity during the CPT. The frequency domains of HRV were acquired by the Fourier transform and divided into three parts: very-low-frequency (VLF, 0–0.04 Hz), low-frequency (LF, 0.04–0.15 Hz), and High Frequency (HF, 0.15–0.4 Hz). The LF/HF was thought to represent the balance of activity of the sympathetic and parasympathetic nervous system, especially the activity of SNS^22^. In our study, after the CPT, the significantly increased LF/HF was found, which indicated that the sympathetic nervous system was activated during the CPT.

The autonomic nervous system maintains the physiological functions of numerous body organs, such as the heart, lungs and gastrointestinal tract^23^. The ocular response for the ANS mainly included the variation of pupil size, intraocular pressure and ocular blood flow. Pupillary size might be influenced by many factors, such as illumination intensity, wakefulness, emotional state etc., but mainly reflected the balance between the tonus of sympathetic (pupil dilatation) and parasympathetic (pupil constriction) nervous system. In our study, pupil diameter was found significantly dilated after cold stimulation, which means that the balance disorders of peripheral ANS occurred. IOP was found to obviously decreased during the cold pressor. The balance of aqueous humor production and outflow determines IOP. Previous studies discussed that parasympathetic and sympathetic innervation involved influenced both aqueous humor production and outflow. But the precise underlying mechanism of any observed changes in IOP is still unclear^24^.

Schlemm’s canal is a vessel that collects aqueous humor and passes it into the bloodstream, thus serving a critical role in the maintenance of stable intraocular pressure (IOP), which is essential for maintaining the eye’s physiological functions^25^. By using a morphometric analysis system, Allingham RR *et al*. found that reduction in SC dimensions in POAG eyes may account for approximately half of the decrease in outflow facility^26^. The SC has attracted considerable interest in recent years^27^. In this study, SCAR was found expanded and was correlated with the activation of SNS (LF/HF index). In our previous work, SNS was stimulated by the aerobic exercise, consequently causing the expansion of the SC lumen and IOP reductions^28^. Histological studies have been revealed that β2-adrenergic receptors expressed in the SC. Specifically, Zhou *et al*. also found that endothelial SC cells softened to isoproterenol via its action on β2 adrenergic receptors^29,30^. Collectively, these findings indicated that the SNS is involved in SC regulation. Significant increases in SCAR following cold stimulation in the present study could be explained by the activation of the SNS.

Mathematical modeling revealed that the dilation of the SC increases its outflow facility and leads to subsequent IOP reductions^31^. An *in vitro* study found that increased IOP was often accompanied by occlusions to the SC lumen^32^. Increasing the length of the SC also improves its outflow, as observed after treatment with SC scaffolds^32,33^. By means of SC-relevant anti-glaucoma surgery, such as canaloplasty, viscocanalostomy, and SC scaffolding, significant decreases in IOP can be achieved^34^. Above all, there’s the close relationship of Schlemm’s canal with the maintenance of stable IOP. In the present study, IOP was found to significantly decrease. And the variation of SCAR and IOP correlated significantly in the post-CPT period. Thus, the IOP trough following CPT might arise from the expansion of SC lumen.

There is a significantly decreased vessel density of retinal tissue after cold stimulation. In 1973, the efficient auto regulation of retinal blood flow was first proposed by Alm and Bill. Any elevation in retinal perfusion pressure is compensated by vasoconstriction in the retinal vasculature to maintain the stability of the retinal blood flow and vice versa^35^. Nagaoka T also found that the constriction of the retinal arterioles plays an important role in the maintenance of retinal blood flow in response to an acute increase in systemic BP using a laser Doppler velocimetry system^36^. The acute increase in MOPP may explain the decreased superficial and deep retinal vessel density in our study.

However, in our observation, there is no significant change in vessel density of optic disc after the activation of SNS. According to Takayama J, the velocity of ONH does not differ significantly between eyes with and without sympathetic nerve amputation; suggesting that the sympathetic system has no effect on ONH circulation^37^. The result is the same as what we found. Many studies have found that the trabecular sheets mechanically distend into the SC when an IOP of 30–50 mmHg is achieved, reducing the SC lumen^38^. However, in this study, we found no significant change in TMTH (trabecular meshwork thickness) following cold stimulation. Thus, the IOP decrease in this study is likely not due to variations of the TM.

While it offers some interesting insights into ocular physiology, the present study also has some limitations that warrant discussion. First, the results might be compared to those in abnormal, aged glaucoma populations or developmental glaucoma populations in future studies. Second, animal models might allow for more precise testing of mechanism and third, using the CPT with different models of ocular degeneration might allow further elucidation of ocular physiology.

## Patients and Methods

### Participants

A total of 24 healthy volunteers from Tongji Hospital in Wuhan (Hubei province, China) were recruited between May 2018 and August 2018. All participants underwent an ophthalmic examination. The eye ipsilateral to the dominant hand was used for all studies.

Inclusion criteria were as follows: 1) IOP < 21 mmHg and normal ophthalmoscopic appearance of the optic nerve (cup-to-disc ratio < 0.5 in both eyes, cup-to-disc ratio asymmetry <0.2, absence of hemorrhage, and localized or diffuse rim thinning); 2) greater than 18 years of age; 3) no history of use of medications that affect the circulatory system within the month prior to their enrolment; 4) no caffeine consumption for at least 24 hr prior to their participation.

Exclusion criteria were as follows: 1) Best-corrected visual acuity (BCVA) < 0.5 (to ensure that the subjects had good central fixation); 2) Refractive error (RE) ≤ −6.0 D and RE ≥ + 3.0 D; 3) serious cataracts or other ocular diseases that hamper optical coherence tomography (OCT) imaging; 4) presence of other eye diseases such as age-related macular degeneration, retinal detachment, or a history of previous eye operations; 5) history or current hypertension or diabetes, or a family history of these conditions.

The study protocols were approved by the ethics committee of Tongji Hospital. Written informed consent was obtained prior to enrollment from all the participants in accordance with the tenets set forth in the Declaration of Helsinki.

### Experimental procedure

#### The experimental group

The experimental recordings were initiated after a 5-min rest period. A baseline recording was obtained (T0, 5 min); followed by a 1-min CPT and 10 min final recovery period (T1: 0–5 min; T2: 6–10 min). For the CPT, the dominant eye and hand were included in this study. Participants were instructed to immerse their hand into an ice water bath (0°C).

#### The control group

The same procedure was repeated on another day. The same subjects were instructed to place their hand into an empty container for 1 min.

Their IOP, BP, HR, PD, ECG recording, the profile images of Schlemm’s canal and trabecular meshwork, the retinal and optic disc angiography images on the same side were acquired successively at T0, T1, and T2.

### Measurement of intraocular pressure (IOP), systolic blood pressure (SBP), diastolic blood pressure (DBP), and heart rate (HR)

Patients fasted for at least 4 hr, after which 3 assessments were conducted. The IOP was measured using an I-care rebound tonometer (I-Care Finland Oy, Vantaa, Finland). All IOP measurements were taken by the same experienced examiner (CW). A different observer (CZQ) read and recorded the IOP readings blinded to minimize any bias.

SBP, DBP, and HR were recorded using an automatic sphygmomanometer (OmronHEM-7201; Omron, Dalian, Liaoning, China). The mean arterial pressure (MAP) and mean ocular perfusion pressure (MOPP) were calculated using the following equation: MAP = [(2 × DBP) + SBP]/3; MMOP = 2/3 (MAP− IOP).

### Measurement of ocular variables

#### Swept-source optic coherence tomography (ss-OCT)

Assessments described in the procedure above were obtained via swept-source optic coherence tomography (OCT) using a 1310-nm wavelength and a scan speed of 30,000 A-scans per sec and an axial resolution of less than 10 μm. 3D-angle high-definition images were obtained using volumetric scans (dimension, a raster of 64 B scans each with 512 A-scans over 8 mm). Nasal and temporal quadrant scans were performed independently, and subjects were instructed to stare at one of two peripheral fixation lights. Conjunctival vessels were used as landmarks to scan the same limbal area before and after cold pressor stimulation. Scans of each site were obtained at three time points (T0, T1, T2) and with the best quality at each position (temporal area and nasal area) was chosen. All OCT tests were performed under standardized darkroom photopic conditions (ca. 3.5 lux).

#### The Angio-Vue optic coherence tomography angiography (Angio-Vue OCTA)

The Angio-Vue optic coherence tomography angiography (OCTA; Optovue Inc, Fremont, CA) system uses a split-spectrum amplitude-decorrelation angiography software algorithm and acquires 70,000 A-scans per sec to create OCTA volumes consisting of 304 × 304 A-scans. A central 6 mm × 6 mm region of the macula and 4.5 × 4.5 mm region of the optic disc were captured. All OCT tests were performed under standardized darkroom photopic conditions (ca. 3.5 lux).

#### Schlemm’s canal cross-sectional area (SCAR) and trabecular meshwork thickness (TMTH)

Schlemm’s canal and trabecular meshwork were imaged during three time-periods (T0, T1, T2) using swept-source OCT (CASIA SS-1000, www.tomey.com) following a 5-min rest period.

**Figure 7:**
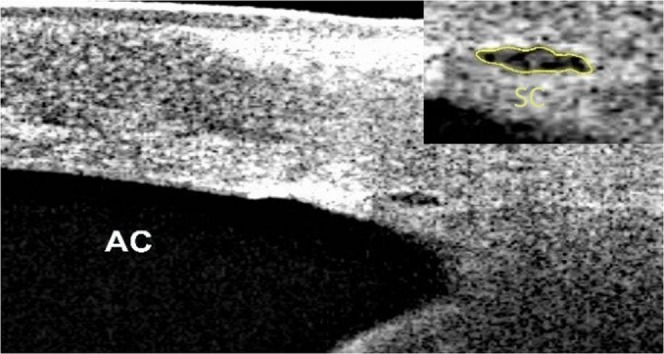
The profile of Schlemm’s canal. The yellow circle shows the profile of the lumen of Schlemm’s canal. AC: Anterior chamber.

The area of the nasal SC (SCAR-n; µm^2^) and temporal SC (SCAR-t; µm^2^) viewed at one region and the trabecular meshwork thickness (TMTH) of two regions (nasal and temporal) at three different time-points were measured using Image J software (version 1.45 S, National Institutes of Health, Bethesda, MD, USA). SC was defined as being observable when a black, lucent space was found (Fig. 7). The thickness of the TM was calculated as the average of measurements acquired at the anterior endpoint and the middle portion of SC, as reported previously^28^.

#### Measurement of retinal vessel density (RVD) and the optic disc vessel density (ODVD)

**Figure 8:**
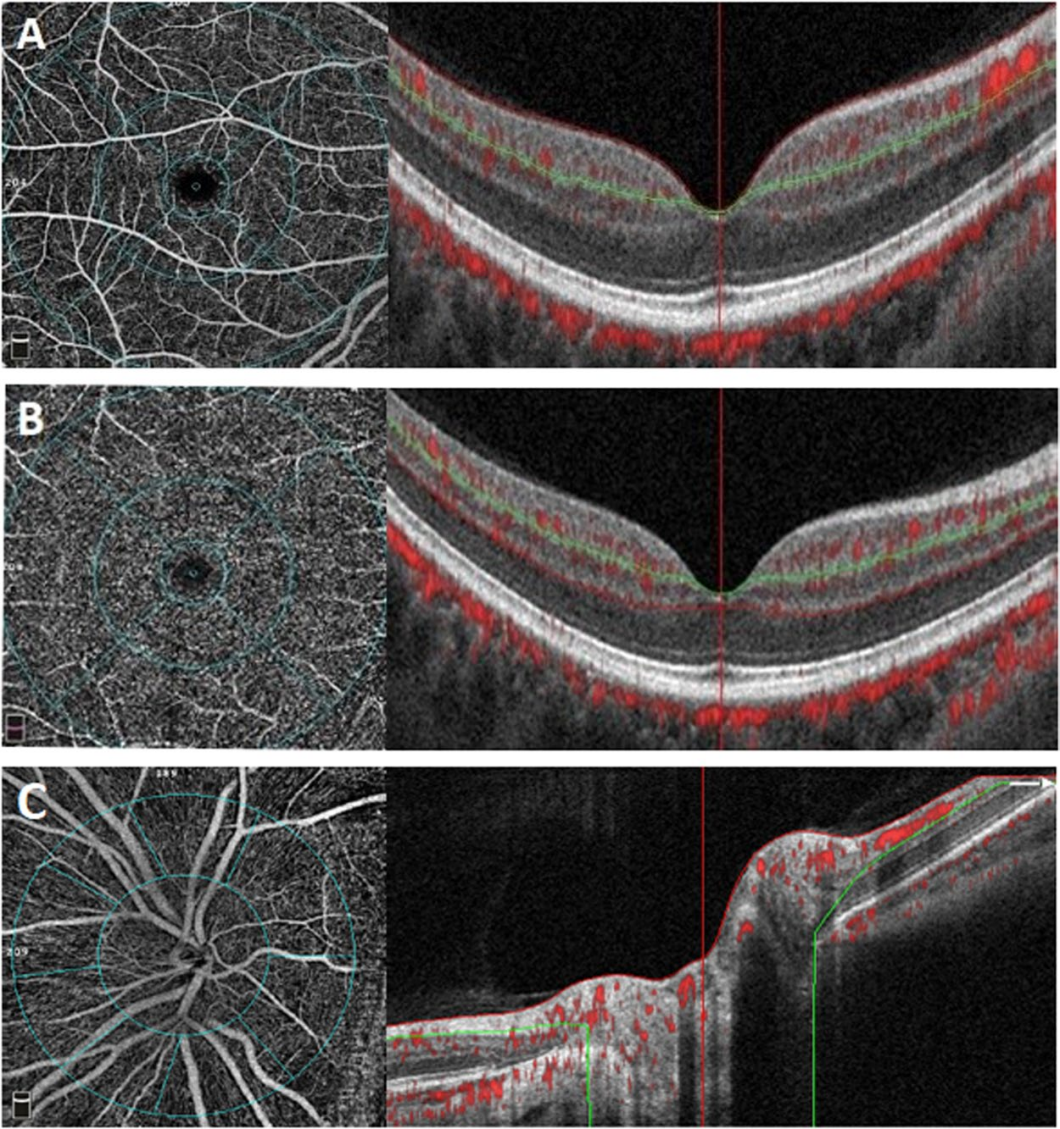
(**A**–**C**) The OCTA images of the macular (6 × 6 mm) and optic disc (4.5 × 4.5 mm) vascular networks. (**A**) The retinal superficial network labeled from 3 mm below the internal limiting membrane to 15 mm below the inner plexiform layer (from red line to green line); (**B**) The retinal deep capillary network marked from 15 mm to 70 mm below the inner plexiform layer. (from green line to red line); (**C**) The Angio disc angiography was performed to generate images (4.5 mm × 4.5 mm) with the optic disc at the center. Optic disc vessel density within the RNFL was measured from internal limiting membrane (ILM) to RNFL posterior boundary (from red line to green line). OCTA: Optic Coherence Tomography Angiography; RNFL: Retinal nerve fiber layer.

The retinal and optic disc vessel density was defined as the proportion of vessel area with blood flow over the total area measured, respectively. The superficial retinal network (s-RVD) included vessels from 3 mm below the internal limiting membrane to 15 mm below the inner plexiform layer; the retinal deep capillary network (d-RVD) included vessels between 15 mm and 70 mm below the inner plexiform layer. Indicators of retinal and optic disc RVD were measured at three time-points around CPT acquisition. (Fig. 8A–C).

#### Measurement of heart rate variability (HRV)

After a 5-min resting period, subjects received the 3-leads ECG recording in the sitting position for 1 min. After the 1 min cold stimulation, the same ECG process was repeated. The R-R peak was extracted and analyzed by the Kubios HRV Premium software (version 2.2; University of Eastern Finland). The index of Low frequency divides high frequency (LF/HF index) was calculated.

All measurements were taken by two observers blinded. Cases of a discrepancy of >15% were resolved by consulting the senior-most author. All data were recorded and stored for later statistical analyses.

### Statistical analyses

All statistical analyses were performed using SPSS software (Version 16.0, SPSS Inc., Chicago, IL, USA). The data are presented as mean values (Means ± SDs). One-way repeated-measures ANOVA was used to compare differences in nasal and temporal SCAR (SCAR-n; SCAR-t), as well as IOP, TMTH, LF/HF, pupil diameter (PD), MOPP, HR, RVD and ODVD at three different time-periods. The paired-sample T test was used to compare the parameters at two different time points in the control group. The linear regression analyses were adopted to examine the relationship between the variations of SCAR-m (the mean value of SCAR-n and SCAR-t) and the change of IOP, the change of PD and the change of LF/HF from T0 to T2 (ΔSCAR-m, ΔIOP, ΔPD and ΔLF/HF). All tests were two-tailed. Statistical significance was defined as a P-value < 0.05.
